# Protection of Antigen-Primed Effector T Cells From Glucocorticoid-Induced Apoptosis in Cell Culture and in a Mouse Model of Multiple Sclerosis

**DOI:** 10.3389/fimmu.2021.671258

**Published:** 2021-06-10

**Authors:** Jasmina Bier, Sebastian M. Steiger, Holger M. Reichardt, Fred Lühder

**Affiliations:** ^1^ Institute for Neuroimmunology and Multiple Sclerosis Research, University Medical Center Goettingen, Goettingen, Germany; ^2^ Institute for Cellular and Molecular Immunology, University Medical Center Goettingen, Goettingen, Germany

**Keywords:** glucocorticoids, apoptosis, T cells, antigen-priming, adoptive transfer EAE

## Abstract

Induction of T cell apoptosis constitutes a major mechanism by which therapeutically administered glucocorticoids (GCs) suppress inflammation and associated clinical symptoms, for instance in multiple sclerosis (MS) patients suffering from an acute relapse. The sensitivity of T cells to GC action depends on their maturation and activation status, but the precise effect of antigen-priming in a pathological setting has not been explored. Here we used transgenic and congenic mouse models to compare GC-induced apoptosis between naïve and antigen-specific effector T cells from mice immunized with a myelin peptide. Antigen-primed effector T cells were protected from the pro-apoptotic activity of the synthetic GC dexamethasone in a dose-dependent manner, which resulted in their accumulation relative to naïve T cells *in vitro* and *in vivo*. Notably, the differential sensitivity of T cells to GC-induced apoptosis correlated with their expression level of the anti-apoptotic proteins Bcl-2 and Bcl-X_L_ and a loss of the mitochondrial membrane potential. Moreover, accumulation of antigen-primed effector T cells following GC treatment *in vitro* resulted in an aggravated disease course in an adoptive transfer mouse model of MS *in vivo*, highlighting the clinical relevance of the observed phenomenon. Collectively, our data indicate that antigen-priming influences the T cells’ sensitivity to therapeutically applied GCs in the context of inflammatory diseases.

## Introduction

Glucocorticoids (GCs) have been used as immunosuppressive and anti-inflammatory agents for more than 70 years (1) and have become indispensable for the management of a variety of diseases. In some fields such as dermatology and ophthalmology, GCs are mostly applied in topical form, whereas systemic administration is customary in the treatment of lymphoma and various autoimmune diseases. This also concerns multiple sclerosis (MS) patients suffering from an acute relapse, who commonly receive high-dose intravenous methylprednisolone pulse therapy to ameliorate clinical symptoms (2).

Most of our current knowledge on the mechanisms of GCs in neuroinflammatory diseases stems from the analysis of experimental autoimmune encephalomyelitis (EAE), a frequently used animal model of MS (3). It has turned out that therapeutic GCs mainly act on T cells in this disease whereas GC nanoformulations such as PEGylated liposomes and hybrid nanoparticles rather target myeloid cells (4–6). GCs engage a variety of mechanisms to modulate T-cell function in neuroinflammation. They alter the expression and secretion of cytokines and chemokines, downregulate adhesion molecules, and redirect T cell migration (4, 5, 7, 8). In addition, they induce apoptosis in developing and mature T cells, which shapes the thymocyte repertoire (9) and contributes to the effectiveness of GC therapy (10). In contrast, the role of regulatory T cells (Treg) as targets of GC action remains controversial (4, 11).

Induction of T-cell apoptosis by GCs requires DNA-binding-dependent transcriptional regulation (12), presumably of pro-apoptotic genes such as Bim and PUMA (13), and is mediated *via* the intrinsic apoptotic pathway (14). The sensitivity of T cells to GC-induced apoptosis depends on their maturation and differentiation stage. While GCs efficiently cause cell death of CD4^+^CD8^+^ double-positive (DP) thymocytes, CD4^+^ and CD8^+^ single-positive (SP) thymocytes turned out to be highly resistant (15–17). Differential sensitivities to GC-induced apoptosis have also been noticed for peripheral T cell subpopulations such as Th1, Th2 and Th17 cells (18), whereas contradictory results have been obtained for Treg cells (19–21). Experiments in cell culture further indicated that activated T cells are less prone to GC-induced apoptosis than naïve T cells. Such a dichotomy was found for both murine (22, 23) and human T cells (24, 25). However, little information is available about the impact of GCs on antigen-specific effector T cells that have been activated in a pathological context *in vivo* rather than by artificial treatment with anti-CD3/CD28 antibodies, PMA/ionomycin or ConA *in vitro*. In order to close this gap, we made use of transgenic mice expressing green or red fluorescent proteins as well as CD45.1/2 congenic mice to distinguish between individual T cell populations, and a protocol to generate antigen-specific effector T cells *in vivo* by immunizing 2D2 transgenic mice expressing a MOG-specific TCR. Using this approach, we could demonstrate that antigen-primed effector T cells are protected from GC-induced apoptosis *in vitro* and *in vivo*, and that this effect is relevant in the context of a model neuroinflammatory disease in mice.

## Materials and Methods

### Mice

C57BL/6J and B6.SJL-PtprcaPepcb/BoyJ (CD45.1-congenic) mice were originally purchased from Charles River (Sulzfeld, Germany) and bred in the animal facility of the University Medical Center Göttingen. The following mouse strains were also housed in the University Medical Center Göttingen animal facility: 2D2 transgenic mice (26), Act-GFP mice (27), and RFP knock-in mice (28). 2D2 and RFP knock-in mice were intercrossed to obtain mice expressing both the MOG-specific 2D2 TCR transgene and RFP. The mice were kept in individually ventilated cages under specific-pathogen-free conditions and supplied with food and water *ad libitum*. All animal experiments were performed in accordance with ethical standards of animal welfare and approved by the responsible authorities of Lower Saxony (*Niedersächsisches Landesamt für Verbraucherschutz und Lebensmittelsicherheit*).

### Animal Experimentation

Mice were immunized s.c. with 50 µg MOG_35-55_ in Complete Freund’s Adjuvant (CFA) and injected twice with 200 ng pertussis toxin (PTX) i.p. on day 0 and 2 as described previously (4). The draining lymph nodes were collected on day 12 post immunization in the case of C57BL/6J mice and on day 10 post immunization for 2D2/RFP mice. To investigate GC effects *in vivo*, 2.5 x 10^6^ restimulated RFP^+^ antigen-primed T cells and 8-10 x 10^6^ purified GFP^+^ naïve T cells were injected i.v. into the tail vein of CD45.1-congenic C57BL/6 recipient mice. Three days later, 5 mg/kg or 20 mg/kg Dex (Urbason soluble 32 mg; Sanofi, Paris, France) or PBS as a control were injected i.p. On day 4 after cell transfer, inguinal and mesenteric lymph nodes and spleen were collected, single cell suspensions prepared and analyzed by flow cytometry.

### T-Cell Isolation and Cell Culture

CD3^+^ T cells were isolated from lymph nodes and spleen with the EasySep™ Mouse T Cell Enrichment Kit (Stemcell Technologies, Cologne, Germany) according to the manufacturer’s protocol. Cell purity was routinely determined by flow cytometry and always exceeded 90%. Antigen-specific restimulation of T cells was achieved by seeding cell suspensions from draining lymph nodes of immunized animals in 96-well round bottom plates at 5 x 10^5^ cells/well in 100 µL of restimulation medium consisting of advanced RPMI 1640 (Gibco 51800-035; Thermofisher Scientific, Darmstadt, Germany), 10% fetal calf serum (PAA Laboratories A15-043; Thermofisher Scientific), 1% penicillin/streptomycin (Gibco 150063), 2 mM L-glutamine (Gibco 25030-024), and 0.0002% β-mercaptoethanol. In addition, 25 µg/mL MOG_35-55_ peptide (wildtype T cells) or 10 µg/mL MOG_35-55_ peptide (2D2 T cells) provided by the Charité (Berlin, Germany), 25 ng/mL recombinant mouse IL-12 (R&D Systems, 419-ML; Wiesbaden, Germany) and 20 µg/mL anti-IFNγ antibody (XMG1.2, Biolegend, 505847; Uithoorn, The Netherlands) were added. The cell suspension was incubated at 37 °C and 5% CO_2_ for 48 hrs, after which it was transferred into 96-well flat bottom plates. Dex at concentrations ranging from 10^-9^ M to 10^-6^ M or restimulation medium alone as a control were added to the wells, followed by 24 hrs incubation at 37 °C and 5% CO_2_.

### Flow Cytometry

All antibodies and reagents for extracellular staining were obtained from BioLegend unless otherwise indicated: anti-CD3ε (145-2C11), anti-CD4 (RM4-5), anti-CD8α (53-6.7), anti-CD19 (6D5), anti-CD44 (IM7), anti-CD45.1 (A20), anti-CD45.2 (104), anti-CD62L (Mel14), AnnexinV, streptavidin, and LIVE/DEAD stain (Thermofisher Scientific). Antibodies and reagents were directly conjugated with FITC, AF647, PE, PerCPCy5.5, PE-Cy7, APC, APC-Cy7, BV421, BV650, BV785 or biotin. Extracellular staining was performed as previously described (29). For intracellular antigen detection, cells were fixed with 4% PFA for 20 min after completion of surface staining, followed by 30 min of incubation with Perm buffer (BD Biosciences, Heidelberg, Germany) and 60 min with an anti-Bcl-2 antibody (clone REA356, conjugated with APC; Miltenyi Biotec, Bergisch Gladbach, Germany), an anti-Bcl-X_L_ antibody (clone 7B2.5, conjugated with biotin; SouthernBiotech, Birmingham, AL, USA) in combination with streptavidin, an anti-Bim antibody (polyclonal, conjugated with AF647, antibodies-online, Aachen, Germany) or an mIgG2b isotype control antibody (clone MPC-11, conjugated with APC or biotin; BioLegend), all diluted in Perm buffer. The mitochondrial membrane potential was measured with the help of the MitoProbe™ TMRM Kit (Thermofisher Scientific) according to the manufacturer’s instructions. All measurements were performed with a Cytoflex^®^ flow cytometry device (Beckmann Coulter, Krefeld, Germany) and analyzed using Cytexpert^®^ (Beckmann) or FlowJo^®^ (Tree Star, Ashland, OR, USA; version 10.7.1.) software.

### Transfer EAE

Lymph node cells from immunized C57BL/6J mice were cultured as described above in the absence or presence of 10^-6^ M or 10^-7^ M Dex for the last 24 hrs of the culture period. Afterwards, 2.5 x 10^6^ cells were transferred i.p. into C57BL/6J recipient mice (30). The induction of transfer EAE did not require application of PTX. Animals were monitored daily for clinical symptoms by measuring their weight and scoring disease progression as follows: 0 = normal; 1 = reduced tone of tail; 2 = limp tail, impaired righting; 3 = absent righting; 4 = gait ataxia; 5 = mild paraparesis of hindlimbs; 6 = moderate paraparesis; 7 = severe paraparesis or paraplegia; 8 = tetraparesis; 9 = moribund; 10 = death.

### Statistical Analysis

All data were analyzed by One-way ANOVA followed by a Newman-Keuls Multiple Comparison test. Analyses were performed with GraphPad Prism software (San Diego, CA, USA). Data are depicted as scatter dots plots with individual data points and a horizontal line representing the mean, or as XY plot with the mean ± SEM. Levels of significance are as follows: n.s., p > 0.05; *p < 0.05; **p < 0.01; ***p < 0.001.

## Results

### Antigen-Priming Protects T Cells From GC-Induced Apoptosis

**Figure 1:**
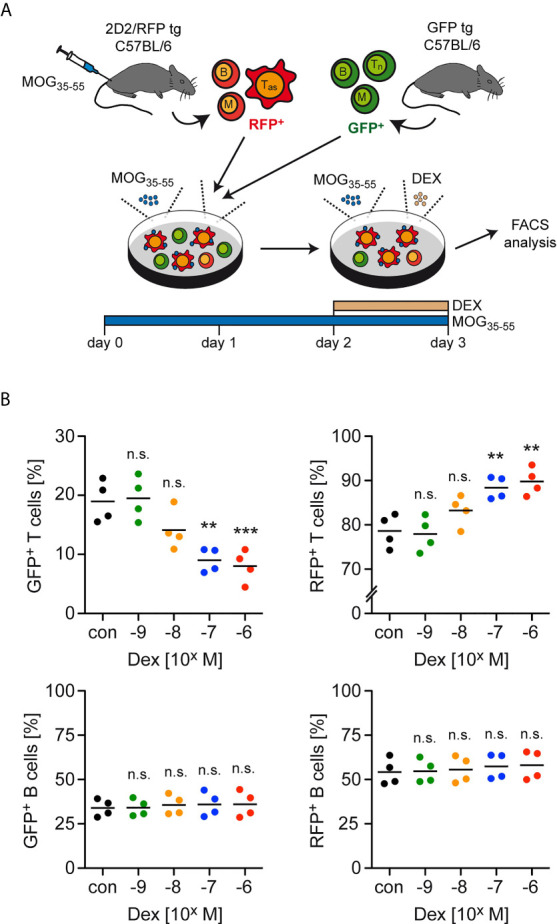
Enrichment of antigen-primed effector T cells after GC treatment *in vitro*. **(A)** Setup of the experimental model. **(B)** Percentages of live/dead^–^ (naïve) GFP^+^ T cells (upper left panel) as well as live/dead^–^ (antigen-primed) RFP^+^ T cells (upper right panel) after 3 days of cell culture in the presence of the indicated concentrations of Dex for the last 24 hrs. In control (con) conditions, no Dex was added to the cell culture. Percentages of GFP^+^ B cells (lower left panel) and RFP^+^ B cells (lower right panel) were determined in the same cultures. Each dot corresponds to cells originating from one individual mouse, the horizontal line represents the mean. N=4 (biological replicates from individual mice). Statistical analysis was performed by One-way ANOVA followed by a Newman-Keuls Multiple Comparison test. Levels of significance (depicted for the comparison between control and Dex-treated cultures): n.s. = not significant; **p < 0.01; ***p < 0.001. T_as_, antigen-specific T cells; T_n_, naïve T cells; B, B cells; M, myeloid cells.

GC therapy of MS patients and EAE mice induces T cell apoptosis but the differential sensitivity of antigen-primed effector and naïve bystander T cells to the pro-apoptotic activity of GCs has not been explored. To address this issue, we initially established an *in vitro* model that mimics the situation encountered in the treatment of neuroinflammatory diseases (**Figure 1A**). Lymph node cells mostly composed of antigen-primed T cells with a MOG-specific TCR were isolated from 2D2/RFP double-transgenic C57BL/6 mice previously immunized with MOG_35-55_ peptide. These cells were then co-cultured for 3 days in the presence of the same antigen with lymph node cells from GFP transgenic mice containing naïve T cells expressing a natural TCR repertoire. During the last 24 hours of co-culture, Dex was added at concentrations ranging from 10^-9^ M to 10^-6^ M to induce apoptosis while leaving T cell differentiation largely unaffected. Flow cytometric analysis revealed that the abundance of naïve GFP^+^ T cells amongst live cells was gradually diminished with ascending concentrations of Dex, whereas the percentage of antigen-primed RFP^+^ effector T cells was concomitantly increased (**Figure 1B**). In contrast, no such effect was observed for other cell types also contained in these lymph node cell preparations. More specifically, the percentages of GFP^+^ and RFP^+^ B cells amongst live cells for instance remained unaltered after the addition of Dex (**Figure 1B**). We conclude that antigen-primed effector T cells are more resistant to GC-induced apoptosis than naïve T cells.

### Reduced Sensitivity of Antigen-Primed T Cells to Apoptosis Induction

**Figure 2:**
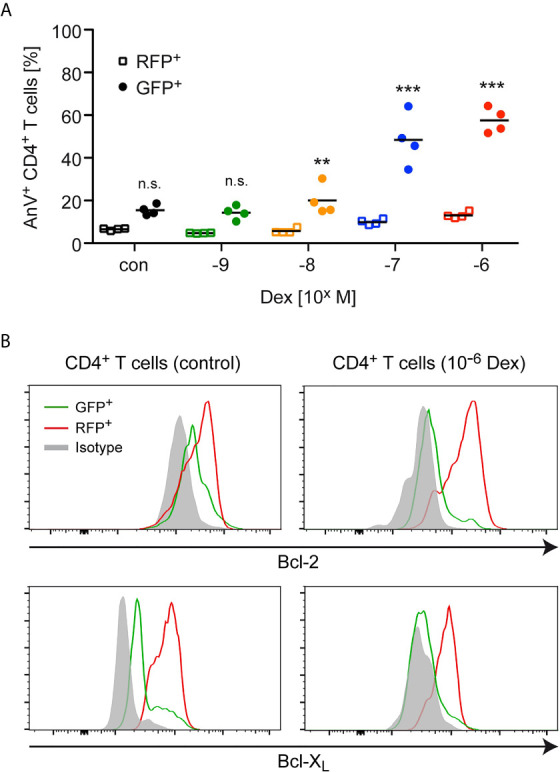
Apoptosis sensitivity of naïve and antigen-primed effector T cells. **(A)** Percentages of AnnexinV^+^ (AnV^+^) cells amongst (naïve) GFP^+^ and (antigen-primed) RFP^+^CD4^+^ T cells after 3 days of cell culture in the presence of the indicated concentrations of Dex for the last 24 hrs. No Dex was added to the cell culture under control (con) conditions. The experimental setup corresponds to the one illustrated in **Figure 1A**. Open symbols refer to RFP^+^ cells and filled symbols refer to GFP^+^ cells. N=4. Statistical analysis was performed by One-way ANOVA followed by a Newman-Keuls Multiple Comparison test. Levels of significance (depicted for each comparison between RFP^+^ and GFP^+^ cells): n.s. = not significant; **p < 0.01; ***p < 0.001. **(B)** Intracellular stainings of Bcl-2 or Bcl-X_L_ in (naïve) GFP^+^ and (antigen-primed) RFP^+^CD4^+^ T cells cultured in the absence (control) or presence of 10^-6^ M Dex for the last 24 hrs according to the experimental scheme depicted in **Figure 1A**. Staining with an mIgG2b antibody served as an isotype control. One representative analysis for each condition is depicted as overlayed histograms.

Next, we explored whether the enrichment of antigen-primed effector T cells after Dex treatment was a direct consequence of an altered sensitivity to apoptosis induction. Using the same experimental setup as before, we found that the percentage of AnnexinV^+^ cells amongst GFP^+^CD4^+^ naïve T cells strongly increased in a dose-dependent manner after Dex treatment, whereas the percentage of AnnexinV^+^ cells amongst RFP^+^CD4^+^ effector T cells increased only very moderately (**Figure 2A**). To further unveil the mechanisms underlying the T cells’ differential sensitivity to GC-induced apoptosis, we investigated the expression of Bcl-2 and Bcl-X_L_ in naïve GFP^+^CD4^+^ and antigen-primed RFP^+^CD4^+^ T cells. Flow cytometric analysis revealed that antigen-priming increased the levels of both anti-apoptotic proteins compared to the naïve state regardless of whether the cells were treated with Dex or not (**Figure 2B**). In contrast, the levels of the pro-apoptotic molecule Bim were comparable in both cell types (data not shown). Therefore, the elevated expression of anti-apoptotic proteins presumably explains the observed protection of effector T cells from GC-induced apoptosis.

**Figure 3:**
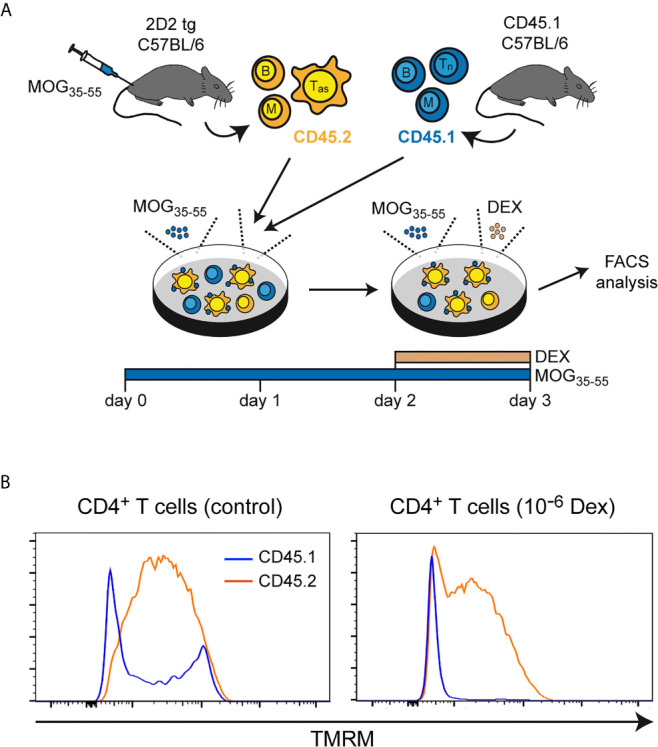
Mitochondrial membrane potential in naïve and antigen-primed effector T cells. **(A)** Setup of the experimental model. **(B)** Intracellular stainings of (naïve) CD45.1^+^ and (antigen-primed) CD45.2^+^CD4^+^ T cells with tetramethylrhodamine methylester (TMRM) after culturing the cells in the absence (control) or presence of 10^-6^ M Dex for the last 24 hrs. One representative analysis is depicted for each condition as overlayed histograms. T_as_, antigen-specific T cells; T_n_, naïve T cells; B, B cells; M, myeloid cells.

To corroborate our findings, we also measured the mitochondrial membrane potential, as Bcl-2 family members have been reported to exert their apoptosis-modifying activity *via* this organelle (31). Lymph node cells, mostly composed of antigen-primed T cells with a MOG-specific TCR, were isolated from 2D2 transgenic C57BL/6 mice previously immunized with MOG_35-55_ peptide. These cells were co-cultured for 3 days in the presence of the same antigen with lymph node cells from CD45.1-congenic mice containing primarily naïve T cells. During the last 24 hours of co-culture, the cells were either treated with 10^-6^ M Dex or left untreated (**Figure 3A**). Under control conditions, a more pronounced loss of the mitochondrial membrane potential, as indicated by a reduced staining with TMRM, was observed for naïve CD45.1^+^CD4^+^ T cells in comparison to antigen-primed CD45.2^+^CD4^+^ T cells (**Figure 3B**). This difference was even stronger after Dex treatment and correlated well with the expression levels of the anti-apoptotic molecules Bcl-2 and Bcl-X_L_ in these cells. Collectively, our results underscore that naïve T cells are the preferential target of apoptotic cell death after GC treatment.

### Enrichment of Antigen-Primed T Cells Coincides With Their Activation State

**Figure 4:**
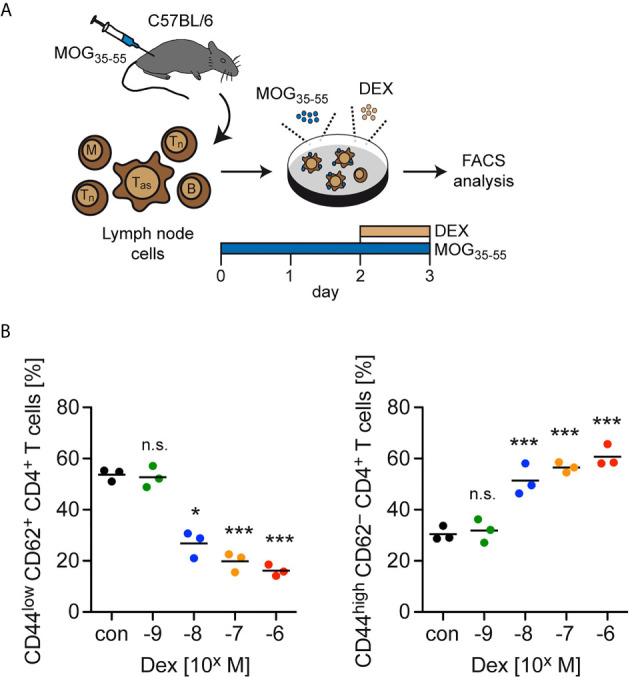
Enrichment of T cells with an activation/memory phenotype after GC treatment *in vitro*. **(A)** Setup of the experimental model. **(B)** Percentages of live/dead^–^ (naïve) CD44^low^CD62L^+^ CD4^+^ T cells (left panel) and live/dead^–^ (activated) CD44^high^CD62L^–^ CD4^+^ T cells (right panel) after 3 days of cell culture in the absence (con) or presence of the indicated concentrations of Dex for the last 24 hrs. Each dot corresponds to cells from one individual mouse, the horizontal line represents the mean. N=3 (biological replicates from individual mice). Statistical analysis was performed by One-way ANOVA followed by a Newman-Keuls Multiple Comparison test. Levels of significance (depicted for the comparison between control and Dex-treated cultures): n.s., not significant; *p < 0.05; ***p < 0.001. T_as_, antigen-specific T cells; T_n_, naïve T cells; B, B cells; M, myeloid cells.

In the last step, we investigated late activation/memory markers as an alternative strategy to distinguish between naïve and effector T cells. Here, we used total lymph node cells from C57BL/6 wildtype mice previously immunized with MOG_35-55_ and restimulated them for 3 days in cell culture. During the last 24 hours, Dex was added at ascending concentrations ranging from 10^-9^ to 10^-6^ M (**Figure 4A**). In agreement with our earlier findings, the abundance of naïve CD44^low^CD62L^+^ cells amongst live CD4^+^ T cells decreased from approximately 60% to 20% with increasing concentrations of Dex, while CD4^+^ T cells with an activated phenotype (CD44^high^CD62L^–^) were concomitantly enriched amongst live cells (**Figure 4B**). In summary, our data suggest that upregulation of anti-apoptotic proteins after activation specifically protects effector T cells from GC-induced apoptosis.

### GC Treatment of Mice Preferentially Induces Apoptosis in Naïve T Cells

**Figure 5:**
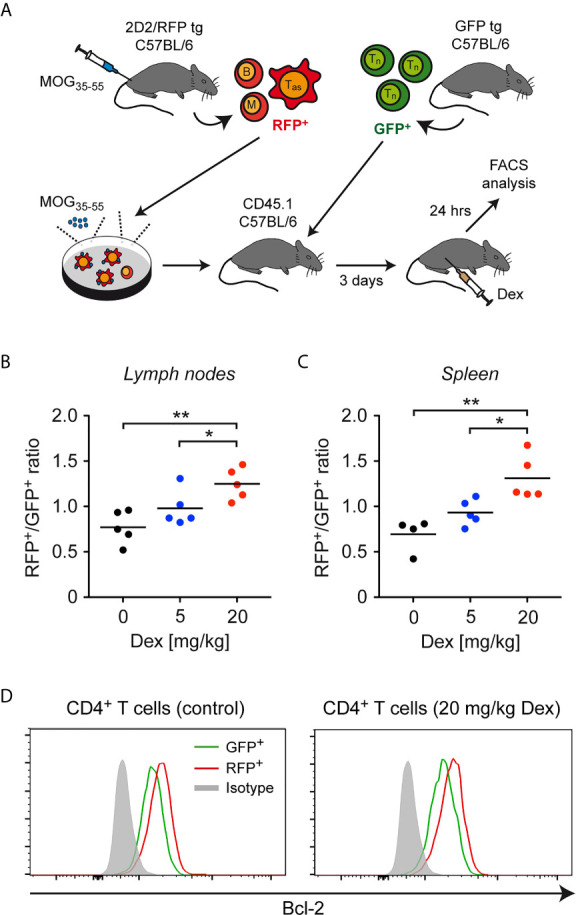
Enrichment of antigen-primed effector T cells after GC treatment *in vivo*. **(A)** Setup of the experimental model. **(B, C)** Weighted ratio of live/dead^–^ CD45.2^+^ (antigen-primed) RFP^+^ T cells and (naïve) GFP^+^ T cells from lymph nodes **(B)** and spleen **(C)** on day 4 after transfer and 24 hrs after application of the indicated doses of Dex (5 mg/kg or 20 mg/kg) *in vivo*. Control mice (0 mg/kg Dex) received PBS. Data are depicted as the mean +/- SEM. N=4-5 (biological replicates from individual mice). Statistical analysis was performed by One-way ANOVA followed by a Newman-Keuls Multiple Comparison test. Levels of significance (depicted for the comparison between control and Dex-treated cultures): *p < 0.05; **p < 0.01. **(D)** Intracellular stainings of Bcl-2 in (naïve) GFP^+^ and (antigen-primed) RFP^+^CD4^+^ T cells isolated from mice treated with 20 mg/kg Dex or left untreated as outlined in panel **(A)**. Staining with an mIgG2b antibody served as an isotype control. One representative analysis for each condition is depicted as overlayed histograms. T_as_, antigen-specific T cells; T_n_, naïve T cells; B, B cells; M, myeloid cells.

As a first step to confirm our results *in vivo*, lymph node cells containing antigen-primed effector T cells expressing a MOG-specific TCR were isolated from 2D2/RFP double-transgenic C57BL/6 mice previously immunized with MOG_35-55_ peptide and restimulated in cell culture with the same antigen. These RFP^+^ effector T cells were then transferred into CD45.1-congenic C57BL/6 mice together with purified GFP^+^ naïve T cells, and 3 days later the mice were injected with 5 or 20 mg/kg Dex or PBS as a control. CD45.1-congenic mice were used as recipients to unequivocally allow to distinguish donor T cells from endogenous cells. After another 24 hours, the ratio between GFP^+^ and RFP^+^ T cells was determined in secondary lymphoid organs by flow cytometric analysis (**Figure 5A**). Similar to our *in vitro* findings, a dose-dependent accumulation of RFP^+^ antigen-primed effector T cells compared to GFP^+^ naïve T cells was observed in lymph nodes and spleen (**Figures 5B, C**). Moreover, also in this *in vivo* setting, a higher expression level of Bcl-2 was found in antigen-primed RFP^+^CD4^+^ T cells than in naïve GFP^+^CD4^+^ T cells regardless of whether or not the cells were treated with Dex (**Figure 5D**). Our data collectively provide evidence that the sensitivity of antigen-primed effector T cells to GC-induced apoptosis in mice is diminished *in vivo* as well.

### GC-Treatment of T Cells Aggravates Adoptive Transfer EAE in Mice

**Figure 6:**
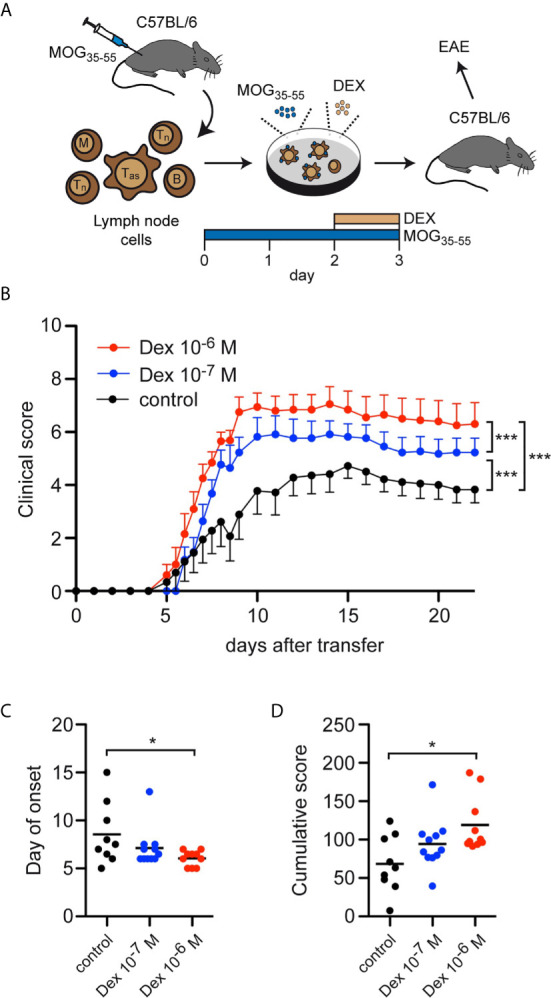
Enrichment of antigen-primed effector T cells after GC treatment *in vitro* aggravates clinical symptoms in an adoptive transfer EAE model. **(A)** Setup of the experimental model. **(B-D)** EAE development after transfer of 2.5 x 10^6^ T cells under control conditions (black) or after treatment with 10^-7^ M (blue) or 10^-6^ M (red) Dex for the last 24 hrs of the cell culture period *in vitro*. Clinical score of all mice that became sick, depicted as the mean +/- SEM **(B)**, the day of disease onset **(C)**, and the cumulative clinical score **(D)** are depicted. N = 11-13. Statistical analysis was performed by One-way ANOVA followed by a Newman-Keuls Multiple Comparison test. Levels of significance: *p < 0.05; ***p < 0.001.

Finally, we asked whether the accumulation of antigen-primed effector T cells after *in vitro* treatment with Dex had any consequences in an adoptive transfer EAE model. To address this issue, C57Bl/6 wildtype mice were immunized with MOG_35-55_ peptide and 12 days later the lymph nodes containing both antigen-primed effector and naïve T cells were isolated and restimulated *in vitro* with the same antigen for 3 days. During the last 24 hours, Dex was added at two different concentrations (10^-7^ M and 10^-6^ M), or the cells were left untreated as a control. Eventually, 2.5 x 10^6^ live cells from each condition were transferred into naïve C57Bl/6 wildtype mice and the disease course was followed over 3 weeks (**Figure 6A**). Mice receiving T cells from cell cultures treated with Dex showed a significantly aggravated disease course dependent on the employed Dex concentration compared to mice receiving control T cells (**Figure 6B**). This negative effect was also reflected by a higher incidence of EAE [10^-6^ M Dex: 91% (10/11), 10^-7^ M Dex: 85% (11/13), control: 75% (9/12)], an earlier onset of the disease (**Figure 6C**), and an increase of the cumulative disease score (**Figure 6D**). In summary, our findings suggest that protection of antigen-primed effector T cells from GC-induced apoptosis is relevant for the pathogenesis of T cell-dependent inflammatory diseases in mice and presumably MS in humans as well.

## Discussion

Induction of T-cell apoptosis was one of the first modes of action identified for GCs and is still believed to be highly relevant for their clinical effectiveness in the treatment of various diseases. It was soon noticed that profound differences exist between individual developmental stages, cellular subsets and differentiation states with regard to apoptosis sensitivity. DP thymocytes are presumably the most sensitive type of immune cell in this respect (16, 17), but differences were also found for mature T-cell subpopulations such as Th1, Th2 and Th17 cells (21, 32). Furthermore, it was observed that T-cell activation conferred protection against GC-induced apoptosis, which was linked to the cell cycle entry into the G1/G2/M phase (33, 34). Along these lines, T cells from burn-injured mice were shown to have a reduced sensitivity to GC-induced apoptosis, which correlated with their enhanced activation status (23). In contrast, very little is known concerning GC-induced apoptosis of antigen-primed effector T cells in the pathological setting of inflammatory diseases. It is noteworthy that a stimulation with anti-CD3/CD28 antibodies, PMA/ionomycin or ConA, as done in most published studies, mimics T cell activation only partially. In natural conditions, activation of T cells is achieved by interaction of an MHC:peptide complex on antigen-presenting cells with the TCR, and engagement of CD28 by B7 molecules in the context of an immune microenvironment composed of other leukocytes, cytokines and growth factors. Since these diverse influences not only impact the fate of effector T cells but presumably determine their sensitivity to GC-induced apoptosis, too, the latter process should be preferably investigated in more sophisticated experimental setups.

To uncover whether antigen-primed effector T cells show a different sensitivity towards GC-induced apoptosis compared to naïve T cells, we developed a co-culture system in which both cell types could be distinguished based on their expression of a red and green fluorescent protein or the expression of the congenic markers CD45.1 and 2, respectively. T cell priming *in vivo* was achieved by immunization of 2D2-transgenic mice expressing a TCR specific for the pathologically relevant myelin antigen MOG_35-55_ followed by restimulation in cell culture. With this setup we ensured that T cell activation occurred in a microenvironment that mimics the situation encountered during an autoimmune response. Importantly, only antigen-specific T cells but neither B cells isolated from the same immunized mice nor naïve T cells were protected from GC-induced cell death. This effect could be assigned to a reduced induction of apoptosis based on the staining of T cell subtypes with AnnexinV and their differential loss of the mitochondrial membrane potential. While these results collectively confirmed that antigen-priming alters apoptosis induction by GCs, this effect was not only clear *in vitro* but also observed *in vivo*. In fact, we could demonstrate that the protective effect of antigen-priming with regard to GC-induced apoptosis was dose-dependent in corresponding concentration ranges *in vitro* (10^-8^ and 10^-7^ M Dex) and *in vivo* (5 and 20 mg/kg Dex). This observation indicates that the mechanism in cell culture and living mice is very similar.

Several mechanisms are being discussed as possible explanations for the diminished sensitivity of activated T cells to GC-induced apoptosis *in vitro*, including a critical role of cytokines as part of the immune microenvironment. This concerns IL-2, IL-4, IL-10 and IL-12 acting on individual T-cell subsets (24, 35), and downstream pathways involving IκBα (36) and PI3K/AKT (37). Importantly, cytokine signaling has been shown to upregulate the anti-apoptotic proteins Bcl-2 and Bcl-X_L_ in activated T cells, which seems to be an important mechanism of their reduced sensitivity to apoptosis induction (38). Intriguingly, Bcl-2 in T cells was found to be only induced after stimulation of total splenocyte preparations with ConA but not after activation of purified T cells with PMA/ionomycin. In contrast, Bcl-X_L_ was induced under both conditions (39). These findings support the notion that the immune microenvironment dictates if anti-apoptotic proteins become elevated during T cell activation or not. In our study, we could show that both proteins were expressed at higher levels in antigen-primed effector T cells compared to naïve T cells, regardless of GC treatment, a finding which reflects the natural context of an autoimmune response *in vivo*. Noteworthy, the previous observation that T cells obtained from Bcl-2-transgenic mice are resistant to GC-induced apoptosis is also in line with our current finding that an increased expression of this anti-apoptotic protein in antigen-primed effector T cells is linked to a protection against cell death by GCs (8). Hence, we consider it fair to conclude that T cells, which were naturally activated in the context of an immune response, become resistant against GC-induced apoptosis through induction of Bcl-2 and Bcl-X_L_.

The biological consequences of the observed GC-resistance of antigen-primed T cells have not been studied in physiologically relevant mouse models up to now. Adoptive transfer EAE mimics many hallmarks of human MS and hence makes it possible to test the *in vivo* relevance of the altered balance between naïve and antigen-primed T cells after Dex treatment *in vitro* (30). Importantly, the MOG-specific T cells analyzed in this study are able to induce clinical symptoms after transfer into C57BL/6 mice and therefore resemble pathogenic T cells found in MS patients. Our results unveiled that the enrichment of antigen-primed effector T cells after incubation with Dex enhanced disease development after adoptive transfer into mice. These data further indicate that our model is very sensitive to the input of antigen-primed effector T cells and that already a slight increase in their relative number suffices to induce a statistically significant worsening of the disease course.

In summary, our study extents previous findings concerning GC-induced apoptosis of activated T cells to the analysis of pathologically relevant antigen-primed effector T cells in a setting which is relevant for neuroinflammatory diseases. The data have several practical implications. Antigen-primed effector T cells can be enriched in cell culture with the help of GCs, which can be exploited for the analysis of autoimmune diseases but also in approaches to treat cancer where pure preparations of such cells are required. Furthermore, the observation that preventive treatment of EAE with methylprednisolone paradoxically worsened the disease outcome can now be explained by an unintended enrichment of effector T cells prior to disease induction (40). Although GCs undoubtedly remain a powerful tool to interfere with inflammatory conditions including MS, our results highlight the importance of critically considering whether their application might result in an enrichment of effector T cells during an ongoing immune response and therefore might even prove harmful.

## Data Availability Statement

The original contributions presented in the study are included in the article/supplementary material. Further inquiries can be directed to the corresponding authors.

## Ethics Statement

The animal study was reviewed and approved by Niedersächsisches Landesamt für Verbraucherschutz und Lebensmittelsicherheit.

## Author Contributions

JB: performed and analyzed experiments. SS: performed and analyzed experiments. HR: designed the project, analyzed experiments, and wrote the manuscript. FL: designed the project, analyzed experiments, and wrote the manuscript. All authors contributed to the article and approved the submitted version.

## Funding

This work was supported by grants from the *Deutsche Forschungsgemeinschaft* (RE 1631/17-1 and FL 377/4-1).

## Conflict of Interest

The authors declare that the research was conducted in the absence of any commercial or financial relationships that could be construed as a potential conflict of interest.
